# Analysis of phenotypic evolution in Dictyostelia highlights developmental plasticity as a likely consequence of colonial multicellularity

**DOI:** 10.1098/rspb.2013.0976

**Published:** 2013-08-07

**Authors:** Maria Romeralo, Anna Skiba, Alejandro Gonzalez-Voyer, Christina Schilde, Hajara Lawal, Sylwia Kedziora, Jim C. Cavender, Gernot Glöckner, Hideko Urushihara, Pauline Schaap

**Affiliations:** 1 College of Life Sciences, University of Dundee, Dundee, UK; 2 Department of Systematic Biology, Uppsala University, Uppsala, Sweden; 3 Department of Integrative Ecology, Estación Biológica de Doñana (EBD-CSIC), Sevilla, Spain; 4 Department of Environmental and Plant Biology, Ohio University, Athens, OH, USA; 5 Department of Genomics, Leibniz-Institute of Freshwater Ecology and Inland Fisheries, IGB, Berlin, Germany; 6 Institute for Biochemistry I, University of Cologne, Cologne, Germany; 7 Faculty of Life and Environmental Sciences, University of Tsukuba, Tsukuba-shi, Japan

**Keywords:** evolution of multicellularity, morphogenetic signalling, phylogenomics, phototropism, encystation, sporulation

## Abstract

Colony formation was the first step towards evolution of multicellularity in many macroscopic organisms. Dictyostelid social amoebas have used this strategy for over 600 Myr to form fruiting structures of increasing complexity. To understand in which order multicellular complexity evolved, we measured 24 phenotypic characters over 99 dictyostelid species. Using phylogenetic comparative methods, we show that the last common ancestor (LCA) of Dictyostelia probably erected small fruiting structures directly from aggregates. It secreted cAMP to coordinate fruiting body morphogenesis, and another compound to mediate aggregation. This phenotype persisted up to the LCAs of three of the four major groups of Dictyostelia. The group 4 LCA co-opted cAMP for aggregation and evolved much larger fruiting structures. However, it lost encystation, the survival strategy of solitary amoebas that is retained by many species in groups 1–3. Large structures, phototropism and a migrating intermediate ‘slug’ stage coevolved as evolutionary novelties within most groups. Overall, dictyostelids show considerable plasticity in the size and shape of multicellular structures, both within and between species. This probably reflects constraints placed by colonial life on developmental control mechanisms, which, depending on local cell density, need to direct from 10 to a million cells into forming a functional fructification.

## 1. Introduction

A central problem in biology is to understand how complex multicellular life forms evolved from unicellular ancestors. In many and perhaps all cases, colony formation may have been the first step towards multicellularity [1]. Although higher plants and animals have converted to zygotic multicellularity, colonial or aggregative multicellularity still occurs in many eukaryote kingdoms, such as Chromalveolata [2], Excavata [3], Amoebozoa [4,5] and Opisthokonta [6].

We investigate molecular changes that allowed colonial organisms to achieve greater levels of multicellular complexity. Dictyostelid social amoebas offer unique opportunities to resolve this problem. They are a genetically diverse group [7], which contains species that form structures of less than 100 cells and one or two cell types to species that can organize up to a million amoebas in a fruiting body consisting of five different cell types [4,8–10]. Over a 100 species have been isolated, which can be subdivided into four major groups based on small subunit (SSU) rRNA and α-tubulin sequence data [11,12]. The genomes of species representing the major groups are now sequenced ([7,13,14]; P. Schaap & G. Glöckner 2013, unpublished data), providing information of genotypic evolution in Dictyostelia. To link this information to evolution of multicellularity, phylogeny-wide phenotypic analysis of Dictyostelia is required. Phenotypic characters were previously collated from original species diagnosis [11]. However, these diagnoses span a period of 150 years and are not consistent in the range, depth and accuracy of character evaluation.

In this work, we have measured 21 traits that were partially covered by the original diagnoses and we investigated deeper traits, such as the alternative survival strategy of encystation, the ability to form motile ‘slugs’ and the identity of the signals that coordinate cell movement during aggregation and morphogenesis. The dataset of phenotypic traits was submitted to phylogenetic comparative analysis to retrace character history and identify correlated evolution of characters.

## 2. Material and methods

### (a) Analysis of phenotypic characters

#### (i) Culture

For analysis of morphology, species were cultured on non-nutrient (NN) agar with pregrown *Klebsiella aerogenes* [15]. For other experiments, species were co-cultured with *K. aerogenes* on one-fifth standard medium or one-third lactose-peptone agar with charcoal for robust and delicate species, respectively (Excel file ‘Trait_Analysis’, sheet 8).

#### (ii) Morphological characters

The shape and dimensions of multicellular structures were mostly assessed or measured *in situ* from agar plates, using a Leica MZ16 stereo microscope, equipped with graticule. The morphological characteristics of amoebas and stalks were assessed by transferring cells and structures to a droplet of phosphate buffer (PB) (10 mM Na/K-phosphate, pH 6.5) on a slide glass. Spore dimensions were measured from printed images. Each feature was measured in 25–50 individuals of each species.

#### (iii) Phototaxis and phototropism

Amoebas were deposited as 10 µl streaks, containing 3 × 10^7^ or 10^8^ cells ml^−1^ on agar and incubated under unilateral illumination. After 1–8 days, the developing structures were scored for phototaxis or phototropism.

#### (iv) Effects of putative chemoattractants on development

Amoebas were resuspended at 10^7^ or 3 × 10^7^ cells ml^−1^ in PB and incubated for up to 36 h as 10 µl droplets on NN agar containing Sp-cAMPS (Biolog, Bremen, Germany), glorin (Phoenix Pharmaceuticals, Burlingame, CA), folate (Sigma) or neopterin (Fluka, Buchs, Switzerland) with the solvents of either compound as controls.

### (b) Phylogenetic comparative analyses

#### (i) Phylogenetic inference

The sequences of 32 orthologous proteins were retrieved from six dictyostelid and three amoebozoan genomes and aligned using M-coffee [16]. The concatenated alignment was used to construct a rooted Dictyostelid core phylogeny with MrBayes v. 3.2 [17] and RaxML in TOPALi [18]. A rooted phylogeny for all Dictyostelia was prepared by Bayesian inference after concatenating the SSU rDNA alignment for all species [12] to the 32 protein alignment. See the electronic supplementary material, figures S4 and S5 for details.

#### (ii) Ancestral state reconstruction

The SSU rDNA_32 protein phylogeny (see the electronic supplementary material, figure S5) was combined with the matrices of continuous or coded categorical characters (sheets 2 and 3 of ‘Trait_Analysis’). For continuous traits, ancestral states were estimated by a maximum-likelihood-based method under a Brownian motion model of evolution [19]. All traits showed high lambda values (range 0.65–0.94), indicating that this model provides an adequate fit to the data (see the electronic supplementary material, table S1). For categorical traits, marginal ancestral states were estimated using a Markov continuous time model [20] in phytools [19]. For each trait, the fit of a model where all transitions were set to equal values (equal probability of gain or loss of the trait) was compared with a model where each transition parameter could take a distinct value. The model that provided a better fit to the data was selected using a likelihood ratio test.

#### (iii) Correlation analyses

Correlated evolution of quantitative characters was determined using phylogenetic generalized least squares (PGLS; [21]), as implemented in the package CAIC [22]. Correlations between all characters (continuous and categorical) were determined using the ‘Discrete’ test [20], implemented in Mesquite [23]. ‘Discrete’ compares a model in which transitions among character states are independent, with a model in which transition rates depend on the state of the other character (correlated evolution). The fit of the models is evaluated using a likelihood ratio test. Significance (*p*-values) of the log likelihood differences were estimated with 100 simulations. To correct for wrongly rejected null hypotheses in multiple comparisons, the threshold *p*-value for rejecting the null hypothesis (no correlation between datasets) was adjusted by a false discovery rate-based method [24].

## 3. Results

### (a) Phenotype analysis

#### (i) The cellular level

**Figure 1.**
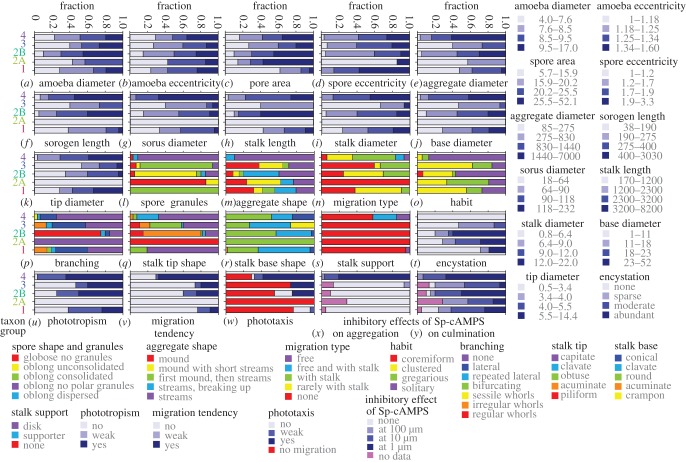
(*a*–*y*) Fractions of character states in each major group or clade. Qualitative and quantitative features that define species phenotype were measured or investigated over 99 species. For quantitative characters, the range of measured values was divided into four intervals, representing the character states. For qualitative characters, the states represent the observed different versions of the feature. The number of states for each character was counted for each major taxon group or clade of the Dictyostelid phylogeny [11] and divided by the number of species per group/clade. For polymorphic characters, the dominant state was counted as 2/3 and the less dominant state as 1/3. The resulting fractions were plotted as stacked bar graphs in which the colours denote the different character states as indicated.

Growing amoebas are morphologically similar in all dictyostelids, and show filose pseudopodia, prominent food vacuoles and contractile vacuoles [9]. Spores, which are formed in fruiting structures, range from round to narrowly elliptical and often contain conspicuous granules at their poles, which are either grouped tightly (consolidated) or loosely (unconsolidated). Continuous (quantitative) characters, such as cross-section area, length and diameter were measured for amoebas and spores, and recalculated to yield average diameter and eccentricity (length divided by diameter). The descriptive statistics of these and all other quantitative traits are summarized in Excel file ‘Trait_Analysis’, sheet 2. The range of averaged values per species was subdivided into four intervals, such that each interval contained an equal number of species. These intervals, the character states, are plotted onto the previously constructed SSU rDNA phylogeny [12] in the electronic supplementary material, figure S1. The states of categorical (qualitative) characters were also plotted onto the phylogeny in the electronic supplementary material, figure S1. To assess group-specific trends in character evolution, proportional representations of character states in each group were calculated (figure 1). Group 2 was subdivided in clade 2A, which contains only acytostelids and clade 2B, which contains a mixture of polysphondylids and dictyostelids. Across most characters, clade 2A was more different from clade 2B than from any other group.

Amoeba diameters range from 4 to 17 µm between species, but there are no marked differences in amoeba dimensions between groups (figure 1*a*,*b*). Spores were markedly smaller in group 1 (figure 1*c*) and usually harboured consolidated granules in groups 1 and 3, and unconsolidated granules in clade 2B (figure 1*l*). Most species in clade 2A have round spores, whereas the oblong spores of group 4 species mostly lack prominent polar granules.

#### (ii) Multicellular structures

Cells can either aggregate as individuals forming a mound, or join up to form inflowing streams, which sometimes fragment into accessory aggregates. Intermediate forms, where cells first aggregate individually and then form streams, or do both, also occur. In group 4, species always aggregate in streams, whereas species in the other groups show a mixture of aggregation types (figure 1*m*). Aggregates are on average larger in groups 4 and clade 2B than in groups 1, clade 2A and 3 (figure 1*e*). Aggregates transform into one or more fruiting body precursors, the sorogens or slugs. Slugs are also relatively large in groups 4 and clade 2B, and this trend continues in the dimensions of the stalk and spore head (sorus) of the fruiting body (figure 1*f–k*). Despite these trends, there is large size variation between multicellular structures within species (see the electronic supplementary material, figure S2), illustrating that species can accommodate greatly varying amounts of cells within a single structure.

Sorogens either form fruiting bodies directly or migrate horizontally, often leaving the stalk behind. Migration is mostly absent or weak in groups 1–3 and common in group 4 (figure 1*n*). Fruiting body habit and branching pattern also show group-specific trends, with group 4 species mainly forming solitary, unbranched fruiting structures, while multiple loosely (gregarious) to tightly (coremiform) grouped fruiting bodies emerge from group 1–3 aggregates (figure 1*o*; electronic supplementary material, figure S1). Lateral branching is common in groups 1 and 3, while most clade 2B species and two species that are intermediate to groups 3 and 4 form regular whorls of branches (figure 1*p*; electronic supplementary material, figure S1).

In most Dictyostelia, the stalk consists of highly vacuolated cells with a cellulose wall, except for clade 2A, where the stalk is a cellulose tube (see the electronic supplementary material, figure S1). The stalk tip is commonly broadened (clavate or capitate) in groups 1 and 4, pointed (acuminate or piliform) in clades 2A and 2B, and a mixture of those in group 3 (figure 1*q*). The stalk base is usually round to conical, but a small clade of group 3 species split the stalk in sections to form a crampon (figure 1*r*; electronic supplementary material, figure S1). Several group 4 species form a cellular basal disc or supporter to buttress the stalk (figure 1*s*).

#### (iii) Encystation

Similar to their amoebozoan ancestors, several dictyostelids can still encyst individually under conditions that do not favour aggregation [9]. To investigate encystation systematically, we exposed species to stress conditions known to trigger encystation. Because cells also died and disintegrated in response to stress, encystation could not be quantitated precisely and we distinguish between sparse (0.1–1%), moderate (1–20%) and abundant (20–100%) encystation. In general, most group 2 polysphondylids and acytostelids encysted abundantly (figure 1*t*; electronic supplementary material, figure S3). One-third of group 1 and half of group 3 species showed sparse to moderate encystation. Remarkably, no group 4 species could be induced to encyst.

#### (iv) Phototaxis and phototropism

Both the migration of slugs and the outgrowth of fruiting bodies are often oriented towards light. These behaviours, termed phototaxis and phototropism, respectively, direct the structures to the soil surface, where spores are readily dispersed [25]. We analysed phototaxis and phototropism by developing species under unilateral light. Phototropism was not observed for acytostelids in clade 2A, but about half of clade 2B species were strongly phototropic (figure 1*u*; electronic supplementary material, figure S1). About 25 per cent of group 1 and 40 per cent of group 3 species showed strong phototropism and over 60 per cent of group 4 species.

Phototaxis requires the ability of slugs to migrate. Migration was also scored during development under incident light (figure 1*n*), but was sometimes worse and sometimes better under unilateral light. In group 4, approximately 70 per cent of species migrate strongly and these species are all phototactic. All migrating group 3 species are phototactic and most of the clade 2B species, but in groups 1 and clade 2A, both migration and phototaxis are weak or absent (figure 1*v*,*w*).

#### (v) Use of chemoattractant

The chemoattractants for aggregation are only known for a few species. *Dictyostelium discoideum* and some group 4 species use cAMP; the *Polysphondylium violaceum* chemoattractant is glorin, a modified dipeptide [26], and the *Dictyostelium minutum* and *Dictyostelium lacteum* chemoattractants were identified as folate and neopterin, respectively [27,28]. Folate is also secreted by bacteria, and attracts many dictyostelids to their food. cAMP additionally coordinates cell movement during fruiting body morphogenesis in *D. discoideum* [29]. Chemoattractants are secreted at nanomolar concentrations, which renders their identification a major challenge. However, a less stringent method can be performed to assess whether known molecules might be used as attractants. When incorporated in the agar at high concentrations, they usually delay or prevent aggregation, by disrupting the natural chemoattractant gradients.

All species were spotted on agar containing increasing concentrations of glorin, folate, neopterin or Sp-cAMPS. The latter was used instead of cAMP to prevent hydrolysis by secreted phosphodiesterases [30]. Developmental progression was scored for deviations from normal aggregation and fructification. Effects on aggregation ranged from time delays, less or smaller aggregates formed, loss of streaming and movement of cells out of the drop. The latter occurs because the cells degrade the chemoattractant inside the drop and move towards the higher concentration outside. If compounds affect aggregation, subsequent multicellular development usually suffers accordingly with less, smaller or no fruiting bodies formed.

**Figure 2.**
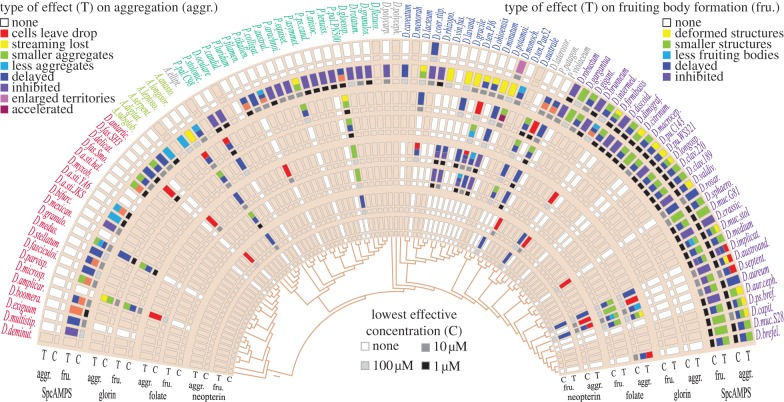
Disruption of aggregation and morphogenesis by putative attractants. Cells were spotted as 10 µl droplets on NN agar containing 1, 10 or 100 µM of either Sp-cAMPS, glorin, folate or neopterin. The progression of aggregation and fructification were recorded at 2 h intervals. The types of deviations (T) from the control treatment (no additives or solvent) that occurred during aggregation (aggr.) and fructification (fru.) are separately shown in the figure by colour-coded boxes. The concentrations where deviations were first observable (C) are shown in shades of grey. Eight delicate species that only develop on charcoal agar could not be tested, because the charcoal adsorbs the attractants. For those species and for chemoattractants that were not tested on all species, boxes retain the beige background colour. The colour-coding of species names reflects group/clade affiliation as in the electronic supplementary material, figure S5.

Sp-cAMPS inhibits aggregation of all group 4 species, but none of the species in groups 1 and 2 (figures 1*x* and 2). Aggregation of a single group 3 species is inhibited and of another promoted by Sp-cAMPS. Strikingly, Sp-cAMPS disrupts or blocks fructification in almost all group 1–3 species (figures 1*y* and 2). The other attractants exert less specific effects on individual species and were, owing to the time-consuming nature of the assays, only tested on four to eight species per taxon group (figure 2). Folate and neopterin affected about half of the species on which they were tested in groups 1, 2 and 4, but mostly only at 100 µM. Folate was particularly effective in disrupting aggregation of over half of group 3 species. None of the tested group 4 species responded to glorin. However, aggregation of *P. violaceum* and its two relatives, *Dictyostelium laterosorum* and *Polysphondylium patagonicum*, which occupy a small clade between groups 3 and 4, was disrupted by 1 or 10 µM glorin, as was aggregation of several species in groups 1–3. There was no effect of folate, glorin and neopterin on culmination that could not be attributed to an earlier effect on aggregation. Asghar *et al*. [31] recently reported that four group 1 and nine clade 2B species showed chemotaxis to glorin, but none of three tested group 4 species. Their choice of species partially overlaps with ours, and both studies confirm each others results.

### (b) Evolutionary analysis

#### (i) Phylogeny reconstruction from 32 proteins

**Figure 3.**
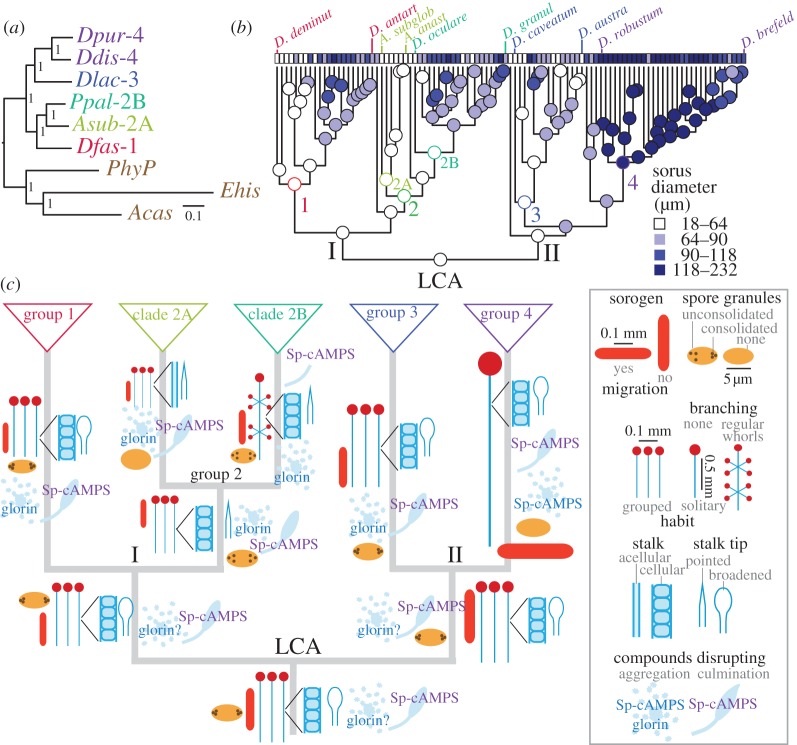
Phylogeny correction and ancestral state reconstruction. (*a*) *Genome-based core phylogeny.* The sequences of 32 orthologous proteins in six group- or clade-representative Dictyostelid taxa (*Ddis*/*Dpur*—group 4, *Dlac*—group 3, *Ppal*—clade 2B, *Asub*—clade 2A, *Dfas*—group 1) and three outgroup taxa *Ehis*, *Acas* and *PhyP* were retrieved from genome sequencing projects, aligned and concatenated. The full alignment and subsets thereof (see the electronic supplementary material, figure S4) were subjected to Bayesian inference for phylogeny reconstruction [17]. Bayesian posterior probabilities of nodes are indicated. Scale bar: number of substitutions per site. (*b*) *Character evolution*. All measured characters were combined with the newly inferred phylogeny for all Dictyostelia (see the electronic supplementary material, figure S5) and subjected to inference of character history and ancestral state reconstruction using maximum-likelihood-based methods. The analysis of quantitative characters is listed in Excel file ‘Trait_Analysis’, sheet 6 and of categorical characters in the electronic supplementary material, figure S6*a–p*. For graphical representation of the evolutionary history of the character ‘sorus diameter’, the range of calculated values was subdivided into four intervals, which, represented by shades of blue, were plotted onto the phylogeny. (*c*) *Ancestral states at major nodes.* For quantitative characters, the state values at nodes that connect major branches (highlighted in colour in ‘Trait_Analysis’, sheet 6) were used to draw fruiting body, slug and spore dimensions at the correct relative sizes onto a schematic of the deep topology of the Dictyostelid phylogeny. Only stalks are presented at one-third of their length, relative to diameter. For all categorical characters that showed a well-defined character history, character states at major nodes were retrieved from the electronic supplementary material, figure S6 and plotted as cartoons onto the phylogeny.

A reliable phylogeny is of primary importance to retrace phenotypic evolution. The earlier SSU rDNA and β-tubulin-based phylogenies subdivided dictyostelids into the same grouping, but did not agree about the root of the tree, with rDNA pointing to group 1 and α-tubulin to group 2 as the earliest diverging group [11]. A phylogeny inferred from 33 proteins from species with sequenced genomes, indicated that the root was placed between groups 2 and 4, but this analysis lacked group 3 sequences and used very distant taxa as outgroup [7]. Meanwhile, three more dictyostelid genomes have been sequenced: *Dictyostelium purpureum* (*Dpur*) from group 4 [14], *D. lacteum* (*Dlac*) from group 3 (http://sacgb.fli-leibniz.de/cgi/index.pl) and *Acytostelium subglobosum* (*Asub*) from clade 2A (http://acytodb.biol.tsukuba.ac.jp/). Additionally, Amoebozoan genomes became available that provide less distant outgroup sequences, such as the genomes of *Entamoeba histolytica* [32], *Acanthamoeba castellani* [33] and *Physarum polycephalum* http://genome.wustl.edu/genomes/view/physarum_polycephalum. We retrieved orthologues for all or most of the 32 genes of the previous set of 33 genes from the six dictyostelid and three amoebozoan genomes and prepared a concatenated alignment of about 18 180 amino acids, which was subjected to different methods for phylogenetic inference. All methods robustly placed the root between two branches that contain groups 1 and 2, and groups 3 and 4, respectively (figure 3*a*; electronic supplementary material, figure S4*a–e*,*o*). This topology was also produced using three out of four concatenated sets of seven of the shorter proteins (see the electronic supplementary material, figure S4*f*,*h*,*i*), but by only two of the four remaining larger proteins (see the electronic supplementary material, figure S4*j*,*m*), while SSU rDNA yielded the same topology (see the electronic supplementary material, figure S4*n*) as previously [11]. The *E. histolytica* sequences diverged strongly from the other amoebozoan sequences, possibly owing to its anaerobic, parasitic lifestyle. Deleting these sequences did not affect the rooting (see the electronic supplementary material, figure S4*e*) and they were omitted from subsequent inference of a phylogeny for all Dictyostelia. This phylogeny was inferred from the earlier alignment of the SSU rDNAs of all species [11,12], concatenated to the 32 protein alignment of the group-representative and outgroup species (see the electronic supplementary material, figure S5). It retains almost the same ordering of species within the four major groups as the earlier SSU rDNA phylogenies [11,12], but the root is now placed between two branches that contain groups 1,2 and groups 3,4. One of two group-intermediate species, *Dictyostelium polycarpum*, which was previously located between groups 2 and 3, is now a sister species to group 2. This is probably not artefactual, since *D. polycarpum* displays morphological features, such as unconsolidated spore granules and pointed stalk tips that are characteristic features of group 2 (see the electronic supplementary material, figure S1).

#### (ii) Ancestral state reconstruction

The newly inferred phylogeny was combined with the characters measured in this work to infer the phenotypes of the last common ancestors (LCAs) of major groupings. Ancestral states for continuous traits were reconstructed using a method based on maximum likelihood [19] and are listed in ‘Trait_Analysis’, sheet 6. The ancestral state values for spore, sorogen and fruiting body dimensions at major nodes of the phylogeny are graphically represented in figure 3*c*. The inference shows that the LCA to all Dictyostelia formed relatively small sorogens and fruiting bodies. The LCAs to groups 1–3 retained their small size, but the group 4 LCA increased fruiting body size about 2.5-fold (figure 3*c*). However, within each group, except for clade 2A, larger forms emerged, as illustrated for sorus diameter in figure 3*b*.

For categorical characters, we estimated ancestral states using a Markov continuous time model [20]. For each character, the proportional likelihoods of ancestral states are plotted as pie sections onto the phylogeny (see the electronic supplementary material, figure S6*a–p*). For several labile characters, such as aggregate and stalk base shape (see the electronic supplementary material, figure S6*b*,*h*), the ancestral states at many major nodes are equivocal, presenting roughly equal likelihoods for all different states. Characters for which ancestral states at major nodes could be inferred with 60–100% probability are summarized in figure 3*c*. The LCA to all Dictyostelia formed unbranched grouped fruiting bodies (see the electronic supplementary material, figure S6*d*,*e*), with a cellular stalk and broadened stalk tip (see the electronic supplementary material, figure S6*g*,*i*). Its spores were elliptical and contained polar granules (see the electronic supplementary material, figure S6*a*). It used cAMP to coordinate fruiting body morphogenesis (see the electronic supplementary material, figure S6*p*), but not aggregation (see the electronic supplementary material, figure S6*o*), which may have been mediated by glorin (see the electronic supplementary material, figure S6*n*; [31]).

Except for the broadened stalk tip, these character states persisted into major branches I and II, and into the LCAs of groups 1–3. The LCA to group 2 gained pointed stalk tips, while the LCA to clade 2A also gained an acellular stalk. The LCA of clade 2B adorned its fruiting bodies with regular whorls of side branches, while the LCA of clade 2A lost polar spore granules. The group 4 LCA also lost polar spore granules, while its sorogens acquired migratory behaviour and its amoebas used cAMP for aggregation. It should be noted that all inference of ancestral states reflects probable trends in trait evolution, which inevitably involves uncertainty, and not a definitive trait history.

#### (iii) Correlated character evolution

To gain initial insight into possible common causes for character evolution and causal relationships between characters, we investigated to what extent changes in individual characters are correlated. Standard statistical correlation methods are not appropriate, because species have varying degrees of shared ancestry and cannot be considered as independent samples [34–36]. To assess correlations between continuous characters, we used PGLS [21], which estimates an evolutionary parameter, lambda, simultaneously with the regression parameters that provides the necessary correction of trait covariance based on the phylogenetic signal of the data [35,36].

The most obvious set of positively correlated traits are the dimensions of aggregates, sorogens and various parts of the fruiting body as the sorus, stalk and stalk base (see the electronic supplementary material, table S2). This is not surprising since large aggregates will normally give rise to large fruiting structures, unless they split up to form multiple sorogens. There is a weak positive correlation between the size of spores and the size of amoebas, and between the size of either amoebas or spores and the size of multicellular structures. The variance in amoeba size explains 12 per cent of the variance in spore size and 4–13% of the size variance in multicellular structures. This suggests that amoeba size contributes to the size of spores and structures, but is not the major determinant.

**Figure 4.**
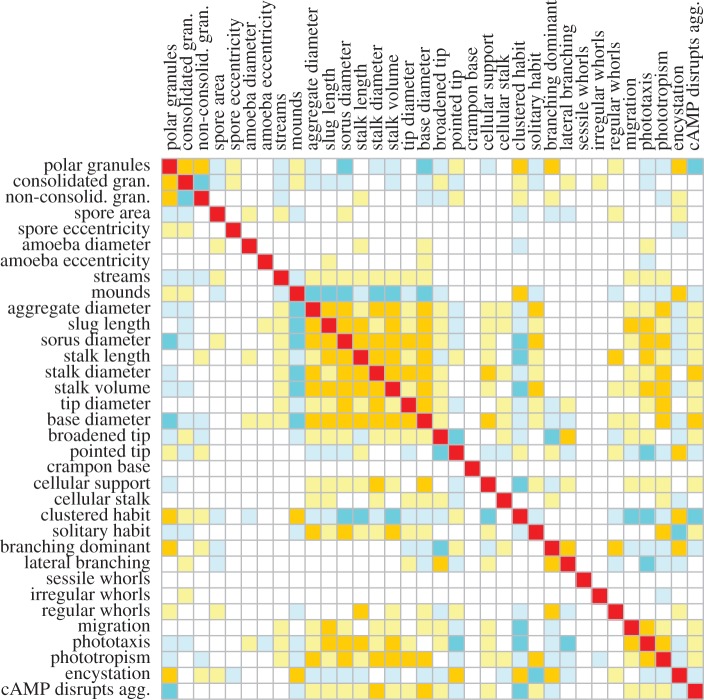
Correlated character evolution. To assess which characters evolved together, the dictyostelid phylogeny was combined with a character matrix that was reconfigured to express all characters in binary form (‘Trait_Analysis’, sheet 4). The ‘Discrete’ correlation test [20] was run with 10 iterations to estimate the log likelihood difference (Likdif) between alternative models of correlated and uncorrelated evolution, and 100 simulations to estimate *p*-values, when appropriate (see the electronic supplementary material, figure S7). Strong positive and negative correlations (*p* = 0; Likdif > 10) are highlighted in amber and dark blue, respectively. Weak positive and negative correlations (*p* < 0.03; Likdif < 10) are highlighted in light yellow and light blue, respectively.

The categorical characters form a much larger dataset and to correlate these characters with each other and with the continuous characters, we used Pagel's ‘Discrete’ test [20], which compares the difference between the log likelihoods of a model where the rates of change in each character are independent of the state of the other and a model where rates of change depend on the state of the other character. The method is only applicable to binary characters, and we therefore transformed multiple and continuous states into binary expressions. In essence, this subdivides continuous characters into two states: small (0) and large (1), while categorical character states become separate characters with states absent (0) and present (1). The correlation matrix is listed in ‘Trait_Analysis’, sheet 7 and is summarized in figure 4.

The strong positive correlations between the dimensions of multicellular structures that were detected with PGLS are also found with ‘Discrete’, indicating that the reduction from continuous to binary character states has no profound effect on the outcome of the analysis. Large size of structures is negatively correlated with a clustered habit, consolidated spore granules and encystation. Mostly, size is not or negatively correlated with dominant branching. However, stalk length is positively correlated with the formation of regular whorls of branches. Interestingly, the size of structures is positively correlated with cAMP-mediated streaming aggregation and with the cellular stalk, slug migration, phototaxis and phototropism, with the latter two features very strongly correlated with each other.

## 4. Discussion

### (a) Robust sporulation from loosely controlled morphogenesis

The traitmap of all measured characters (see the electronic supplementary material, figure S1) shows a fairly scattered distribution of character states over the tree with many states reappearing multiple times in different clades. This is particularly the case for features that describe the size and shape of aggregates, slugs and fruiting bodies. Most species are also individually polymorphic for these characters (see ‘Trait_Analysis’, sheet 3, columns Q–X). Depending on local conditions of cell density, individual species can make aggregates and fruiting bodies in a wide range of sizes. Even when developed under standardized conditions, individual structures from the same species easily show a fourfold difference in size (see the electronic supplementary material, figure S2). At low food availability, even a fairly large species, such as *Polysphondylium pallidum*, can form fruiting bodies from as few as seven cells [8].

The morphology of structures also varies depending on cell density. Species, which normally have clustered or branched fruiting bodies show solitary and unbranched phenotypes when developing from small numbers of cells, while normally solitary and unbranched species show some clustering and branching at high cell density.

There is good evidence that fruiting body morphogenesis in *D. discoideum*, and representative taxa from all four groups is coordinated by cAMP pulses that are emitted by the tips of sorogens and propagate through the structure as standing or spiral waves [29,37–40]. This is likely to be case for all Dictyostelia, since we show in this work that Sp-cAMPS, which desensitizes cells to cAMP pulses, disrupts fruiting body morphogenesis in almost all investigated species (figure 2). cAMP pulses are produced by positive and negative feedback of cAMP on its own synthesis [41,42]. The output dynamics of this network, such as wave form and suppression of competing oscillators, which govern the size and shape of fruiting structures, are easily affected by genetic variation in the component proteins and naturally occurring physico-chemical factors [43–45]. Such variables can account both for morphological variation between species and within species upon exposure to different conditions. This environmentally adaptive system for morphogenetic control and the consequent plasticity of forms that it generates is eminently suited for organizing variable numbers of cells into functional units. However, it contrasts strongly with the superimposed layers of genetic control that shape the body plan of higher animals, where, for obvious reasons, extensive plasticity in the size and shape of organs and appendages is mostly detrimental.

### (b) Trends in the evolution of phenotype

Despite morphological plasticity, there are trends in dictyostelid phenotypic evolution. Ancestral state reconstruction showed that the LCA to all Dictyostelia as well as the LCAs of the two major branches and groups 1–3 probably had small, unbranched fruiting structures, containing elliptical spores with polar granules. cAMP was probably used to coordinate fruiting body morphogenesis, but not aggregation, which could have been mediated by glorin (figure 2; [31]). The LCA to clade 2B evolved fruiting structures with regular whorls of side branches, whereas the group 4 LCA formed large unbranched fruiting bodies. It lost spore granules, but gained cAMP as attractant for aggregation. Slug migration, phototaxis and phototropism evolved several times independently within most major groups.

Correlation analysis highlights coevolution of large, solitary, unbranched multicellular structures, streaming aggregation, slug migration, phototaxis and phototropism, and use of cAMP as attractant (figure 4). The analysis can identify characters that evolved together, but not why this is the case. However, occasionally correlated features can reveal hints into underlying causes. This is, for example, the case for the strong positive correlation between fruiting bodies size and phototropism. This correlation does not only exist between species, but also within phototropic species; smaller fruiting bodies are less phototropic [46]. If phototropism depended on a specialized sensor, there is no reason why small structures should lack phototropism. Bonner *et al*. [47] proposed that ammonia, produced in response to light focused by the tip at the distal side of the slug, locally speeds up cell movement and causes the tip to veer towards the light. This can explain the size dependency of phototropism, since build-up of ammonia in small sorogens would be limited due to dissipation into the atmosphere. Slug migration and phototaxis are also strongly correlated with size between species, and this is also the case within species [48]. Lack of slug migration and light responsiveness could therefore be a consequence of the small size of species.

A more enigmatic relationship that was already noted in the 1970s [49] is the correlation between gain of cAMP as attractant and loss of polar spore granules and encystation. The early workers also associated polar granules with smaller, branched or clustered fruiting structures, as substantiated in this work (figure 4). It can be envisaged that more robust fructification made encystation superfluous, but why this should be connected with spore granules and cAMP is unclear. The answers may come from the function and ontogeny of spore granules and more importantly from the ecological factors that acted on the gain and loss of all these features. These factors, contained in the habitat, geographical origin and local climate conditions of species and their interactions with other organisms in the rhizosphere provide the ultimate cause for phenotypic innovation. However, their influence is at present difficult to address, owing to sparse sampling of most species and limited information on their lifestyles in nature.

The future aim of our work is to identify causal relationships between the evolution of multicellularity and the evolution of genes and genomes. Bioinformatic and experimental approaches are being used to analyse changes in content, regulation and function of developmental control genes between the recently sequenced genomes of species representing all major groups of Dictyostelia. Combined with the opportunity to replace genes in both late and early diverging species with more ancestral or derived alleles, respectively, such approaches have already yielded insight into the evolutionary history of cAMP signalling [50] and can ultimately identify the critical genetic modifications that caused the emergence of multicellular life forms.
